# Self-care and clinical management of persons with laryngectomy during COVID-19 pandemic: a narrative review

**DOI:** 10.1007/s00520-021-06333-3

**Published:** 2021-06-28

**Authors:** Janet Jaison Varghese, Venkataraja U. Aithal, Bellur Rajashekhar

**Affiliations:** Department of Speech and Hearing, Manipal College of Health Professions, Manipal Academy of Higher Education, Manipal, 576104 India

**Keywords:** Laryngectomy, COVID-19, Tracheoesophageal speakers, Alaryngeal speakers, Corona Virus, SARS-COV-2

## Abstract

**Objective:**

To summarize guidelines on self-care and clinical management of persons with laryngectomy during the COVID-19 pandemic.

**Method:**

Articles published in electronic databases—PubMed, Scopus, Web of Science, and CINHAL with the compliant keywords—were scouted from December 2019 to November 2020. All original articles, letters to editors, reviews, and consensus statements were reviewed and included.

**Results:**

In all, 20 articles that had information pertaining to self-care of persons with laryngectomy or guidelines for clinicians working with this population were identified. Four of the included studies were case reports of persons with laryngectomy who contracted the COVID-19 virus. One of the included articles was a cohort study that explored the use of telerehabilitation in persons with laryngectomy.

**Conclusion:**

The hallmarks of preventative strategies for persons with laryngectomy during the COVID-19 pandemic are as follows: physical distancing, use of a three-ply mask or surgical mask to cover the mouth and nose, and use of Heat Moisture Exchange (HME) device over stoma in addition to covering it with a surgical mask or laryngectomy bib. Telerehabilitation, not a preference with this population prior to the pandemic, has gained popularity and acceptance during the COVID-19 situation. The reports of COVID-positive persons with laryngectomy have indicated contrary findings from the tracheal and nasal swabs, necessitating compulsory inclusion of both nasal and tracheal swabs.

## Introduction

The COVID-19 pandemic has resulted in an universal health crisis and has affected all aspects of our lives. COVID-19 (SARS-CoV-2) is manifested with a myriad of symptoms; while some may be asymptomatic-having minimal to no symptoms, others could encounter severe respiratory dysfunction necessitating intensive care and intubation [1].

This virus has been reported to spread by contact, droplets, aerosol, and contaminated environment; there is a higher risk of this disease from those who are symptomatic but asymptomatic individuals can also cause transmission. After an individual contracts the virus, it proliferates rapidly in the respiratory tract and is capable of remaining in surfaces for hours to days. Wearing a mask, social distancing, and hand-hygiene practices are effective ways of reducing the rapid spread of the virus. These guidelines are now the new normal in all countries.

The pandemic has also altered the usual functioning of healthcare systems and workers.

Management of patients with chronic conditions is especially affected during these pandemic times. The COVID-19-related guidelines are given special importance in the case of patients with total laryngectomy in recent literature. It is vital for all clinicians working with laryngectomees to follow these guidelines to ensure superior clinical care and safe society.

Total laryngectomy refers to the surgical removal of the larynx, often done as the last option in those with advanced cancers of the larynx and hypopharynx [2]. It is often preferred when organ preservation protocols have failed or not considered suitable [3]. The surgery results in several anatomical and functional changes. Post-surgery, the individual will be a neck (stoma) breather and no longer capable of producing laryngeal voice [2]. Smell, taste, and swallowing are altered forever [3]. Physical alterations coupled with physiological limitations could lead to psychological issues and social isolation, thereby reducing the individual’s quality of life.

The inability to speak is the greatest concern for majority of the patients. Voice restoration in these individuals is accomplished by the in vogue alaryngeal speech modes viz. esophageal speech, tracheoesophageal speech, and artificial larynx. Out of these three, tracheoesophageal speech is superior considering its acceptability and intelligibility [4]. However, the use of a voice prothesis in this mode can lead to problems and issues of concern. Periodic changing of the device due to wear and tear and other complications like leakage, granulations need to be kept in mind by the rehabilitation team. Though experienced TE speakers would be able to handle some of the minor issues, periodic hospital visits and consultations are unavoidable.

The current COVID crisis presents several challenges for individuals with laryngectomy. Foremost, they are at high risk for droplet transmission diseases such as COVID-19 [5]. After laryngectomy, the contribution of the nose for respiration is limited with its physiological functions of humidifying, heating, and moistening compromised. The presence of a structurally intact nasal cavity and nasopharynx along with a stoma makes the laryngectomee more vulnerable to the disease. The nasal cavity and nasopharynx can house the virus with the stoma providing an additional route for the virus’s entrance [6].

Furthermore, due to the structural alterations and increased chances of aerosolization of tracheal secretions, an individual with a tracheostoma such as a person with laryngectomy could be a risk for his caregivers and community at large [5, 7, 8]. This calls for necessary precautions by family and caregivers to safeguard themselves.

Finally, alaryngeal speakers would need periodic hospital assistance in procuring prostheses and accessories, trouble shooting of devices and complications. In situations such as leakage-related issues, lack of timely attention could result in major health complications such as pneumonia. In the pandemic situation, access to hospitals and clinicians is restricted considering the priority to COVID-19 patients.

Hence, persons with laryngectomy need to be vigilant and cautious in caring for themselves. Clinicians working with them need to protect themselves, offer guidance to individuals with laryngectomy regarding the best precautionary measures, and take decisions regarding patient care and management as per the prescribed policies, given the magnitude of COVID-19 across the globe.

This article summarizes the recommendations for self-care and clinical management of persons with laryngectomy during the COVID-19 pandemic.

## Materials and method

The databases viz. PubMed, Scopus, Web of Science, and CINHAL were consulted from December 2019 to November 2020.The searches were done using MeSH or free text terms combined with Boolean Operators in PubMed; ((“laryngectomy” OR “laryngectomee” OR “laryngectomies” OR “individuals with laryngectomy” OR “Trachesophageal speaker” OR “TE speakers” OR “Tracheo-esophageal speakers” OR “Oesophageal speakers”)) AND ((“COVID-19” OR “covid 2019” OR “severe acute respiratory syndrome coronavirus 2” OR “2019 ncov” OR “sars cov 2” OR “2019ncov” or “wuhan coronavirus”)). This search strategy was modified for other databases as appropriate, mentioned in Table 1.

**Table 1:** Search strategy

S no	Database	Search strategy
1	**Pubmed**	**((“laryngectomy” OR “laryngectomee” OR “laryngectomies” OR “individuals with laryngectomy” OR “Trachesophageal speaker” OR “TE speakers” OR “Tracheo-esophageal speakers” OR “Oesophageal speakers”)) AND ((“COVID-19” OR “covid 2019”OR “severe acute respiratory syndrome coronavirus 2” OR “severe acute respiratory syndrome coronavirus 2” OR “2019 ncov” OR “sars cov 2” OR “2019ncov” or “wuhan coronavirus”))**
2	**WOS**	#1 ALL = (“laryngectomy” OR “laryngectomee” OR “laryngectomies” OR “ individuals with laryngectomy” OR “Trachesophageal speaker” OR “TE speakers” or “ Tracheo-esophageal speakers” OR “Oesophageal speakers”) *Indexes* = *SCI-EXPANDED, SSCI, A&HCI, CPCI-S, CPCI-SSH Timespan* = *All years* #2 ALL = (“COVID-19” OR “covid 2019” OR “severe acute respiratory syndrome coronavirus 2” OR “severe acute respiratory syndrome coronavirus 2” OR “2019 ncov” OR “sars cov 2” OR “2019ncov” or “wuhan coronavirus”) *Indexes* = *SCI-EXPANDED, SSCI, A&HCI, CPCI-S, CPCI-SSH Timespan* = *All years* Combine #1 AND #2
3	**CINAHL**	TX ( (“COVID-19” OR “covid 2019” OR “severe acute respiratory syndrome coronavirus 2” OR “severe acute respiratory syndrome coronavirus 2” OR “2019 ncov” OR “sars cov 2” OR “2019ncov” or “wuhan coronavirus”)) AND TX ( (“laryngectomy” OR “laryngectomee” OR “laryngectomies” OR “ individuals with laryngectomy” OR “Trachesophageal speaker” OR “TE speakers” or “ Tracheo-esophageal speakers” OR “Oesophageal speakers”))
4	**SCOPUS**	ALL ( *“laryngectomy”* OR *“laryngectomee”* OR *“laryngectomies”* OR *“individuals with laryngectomy”* OR *“Trachesophageal speaker”* OR *“TE speakers”* OR *“Tracheo-esophageal speakers”* OR *“Oesophageal speakers”*) AND ALL (*“COVID-19”* OR *“covid 2019”* OR *“severe acute respiratory syndrome coronavirus 2”* OR *“severe acute respiratory syndrome coronavirus 2”* OR *“2019 ncov”* OR *“sars cov 2”* OR *“2019ncov”* OR *“wuhan coronavirus”*)

This study included all original articles, letters to editors, reviews, and consensus statements published in English. Two researchers independently carried out the title and abstract screening of the articles. Both researchers reached a consensus on the studies to be included. In order to make the review more comprehensive, case reports of persons with laryngectomy who tested positive for COVID-19 were also included. Additionally, hand-searching of the reference lists of all included studies and forward searching of all the citations of the selected articles were also done to identify articles that could have been missed out.

As the objective was to cumulate a narrative review, quality appraisal or risk of bias assessment of the included articles was not done. The included studies are categorized under appropriate headings and discussed below.

## Results

### Characteristics of included studies

Searching the databases with the keywords yielded 112 articles; 43 duplicates were removed and 69 articles were considered for title and abstract screening. Eighteen articles were deemed relevant. Fifty-one were excluded as they did not present any relevant information pertaining to persons with laryngectomy, in particular. Forward reference searching was carried out from the selected studies which resulted in identifying 2 articles. Finally, 20 articles were included in the review. All included studies were in the form of correspondence, commentary, review, or letters to the editor. Four out of the twenty articles were case reports and one article was a cohort study on telerehabilitation. All the articles and case reports are summarized in Table 2 and Table 3 respectively.
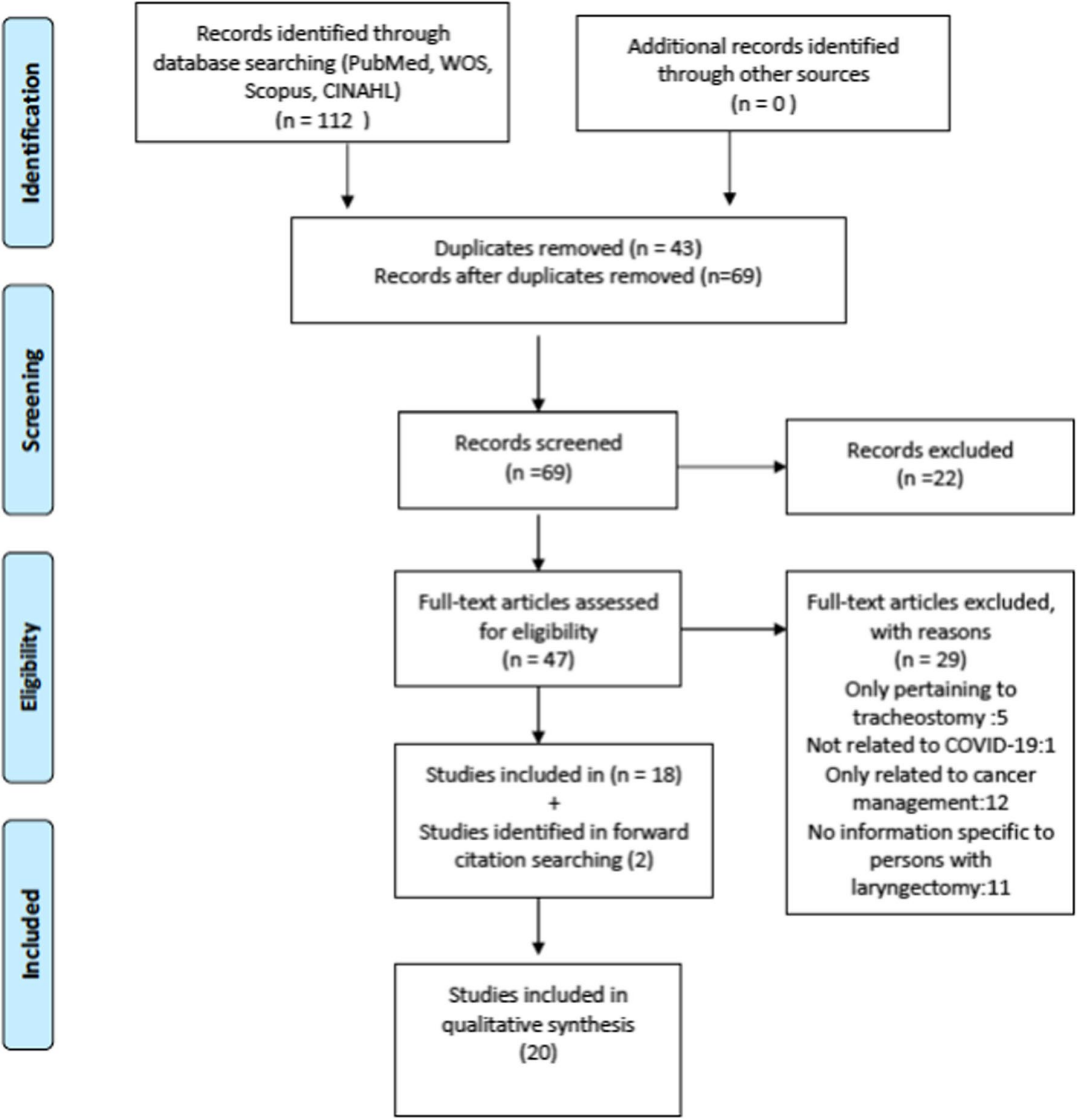

**Table 2:** Suggested precautionary measures for patients and clinicians

Aspects	Suggestions
Guidelines for individuals with laryngectomy	
Self-care and precautionary measures	• Avoid going out as much as possible [18] • Follow social distancing and stringent hand hygiene [6, 12, 18, 27] • Use a 3ply mask or surgical mask to cover the mouth and nose [6, 9, 10, 14, 18, 27] and another mask to cover the stoma [5, 12] • Use of an HME is highly recommended [6], followed by covering it with a surgical mask or laryngeal bib or laryngectomy apron or clothing [5, 6, 9, 10, 14, 15, 18, 27] • Use of hands-free device is advisable [5, 12] or Use of non-dominant hand for stoma occlusion [12] • Use of sanitizer before and after touching the stoma is recommended [18] • Micron HME is the only known virus filter device [9, 12] • Provox Micron HME filter can be used for COVID-19 positive laryngectomees [10]
Device care	• Artificial larynx to be cleaned frequently with alcohol-based wipes [6] • Non-indwelling voice prosthesis and pneumatic device and the cleaning brush of indwelling device to be immersed in disinfectants before cleaning [6] • HME Device must be disposed after community exposure [18]
Guidelines for clinicians working with persons with laryngectomy	
Self-protection	• Use standard PPE, i.e., N95 with face shield, surgical gloves while seeing COVID negative patients [27] • Enhanced PPE while working with COVID-19 positive or suspected COVID-19 patients or unsure of the diagnosis [5, 10, 17] • Regardless of the COVID status, always use enhanced PPE while doing procedures with high risk of generating aerosols [10], i.e., changing the voice prosthesis, leakage inspection, stoma care etc. [9, 15, 16]
Initial screening and triaging	• Use telerehabilitation whenever possible [5, 13] especially for screening purposes. However, also remember that alaryngeal speech may pose a communication barrier in telemedicine [21] • Compare the urgency and seriousness of the issue against risk of hospital visit before asking the patient to visit the hospital [21] • Patients must be advised self- management of leakage-related issues at home whenever possible or until hospital visit [16, 18]
Recommendations to manage prothesis-related leakage at home	• Using thickeners for liquids [13, 15, 17, 18, 20] • Use of plug in their voice prosthesis [13, 17, 20] or catheter until hospital visit [18] • Cleaning prosthesis to ensure valve is proper, cyclic intake of solid and liquid food [13] • Temporarily using the inserter stick as a plug, using cotton tipped applicators to absorb droplets from the sides of the value opening [13] • In case the TE speech fails, AACs and artificial larynx is recommended [20]
Direct consultation	• Delay in the management of leakage-related issues may cause aspiration and breathing difficulties [21]; therefore, hospital visit is unavoidable or cannot be prolonged for long in certain cases • COVID Testing of the patient (both nasal and tracheal swab) to be done before direct trouble shooting of TE related issues [5] • Use of physical barrier such as to create isolated space for physical examination of patient can be used if possible [26] • Use of lidocaine spray to prevent cough [9]
Telerehabilitation	• Telemedicine facilitates access to care [21]; can be used for management of patients as well as surveillance [20] especially those using TE speech [11, 13] • Minor issues can be resolved via video calls and reserve the hospital visit to those who need immediate medical attention [11] • Involve caregiver in the telecall in order to overcome speech barrier [20] • Use educational materials such as videos on stoma care and information on precautionary measures

**Table 3:** Summary of case reports

S no	Title of article	AUTHORS	country	Suggestions/findings
1	“COVID-19 and Total Laryngectomy—A Report of Two Cases”	Paderno et al., 2020	Italy	Laryngectomy patients with COVID-19 manifest tracheal complications due to lack of natural humidification and effect of oxygen therapy. Dedicated HME devices that can be paired with oxygen supply was not available
5	“Disparate Nasopharyngeal and Tracheal COVID-19 Diagnostic Test Results in a Patient With a Total Laryngectomy”	Patel et al.,2020	USA	Contrary findings from nasal and tracheal swab; this indicates the importance of taking both nasal and tracheal swabs for COVID testing
8	“Severe acute respiratory syndrome coronavirus-2 in post-laryngectomy patients: case series of four patients.”	Coleman et al.,2020	Scotland	Case report of four individuals with laryngectomy who tested positive for COVID, all recovered without the need for intensive care
9	“SARS-CoV-2 in upper and lower airway samples of a laryngectomized patient: new insights and many lessons.”	Gallo et al.,2020	Italy	Case of individual with laryngectomy who tested positive in both the nasal and oropharyngeal swab, and further confirmed with tracheal swab

### Guidelines for persons with laryngectomy:

#### Self-protection of person with laryngectomy

Wearing a mask, hand hygiene practices, and social distancing measures are the universal precautionary measures to be followed during the pandemic. In persons with laryngectomy, the presence of an additional orifice in their neck with direct conduit to the trachea necessitates an additional covering of their stomas. Laryngectomy bib or high neck clothing may serve the purpose [9], but the use of a surgical mask to cover the stoma is highly recommended [5, 10]. Furthermore, it is advised to tie it in the following manner- upper strings tied around the neck and using an extender string to tie the lower strings at the back [11, 12]. Usage of N95 mask is not recommended as it does not provide an adequate seal around the stoma [5]

Covering the stoma with a Heat Moisture Exchange (HME) device offers many advantages [6, 10], with usage of a HME that comprises of a virus filter is the best possible approach [5]. HMEs benefit the patient by filtering the inhaled air and providing humidification. In addition to reducing crusting [13], an added advantage is the reduction of mucous production, cough, and expectoration and the resultant reduction of aerosol and droplet formation [10]. These are important factors, considering the high transmission rate of SARCOV-2 virus through tracheal secretions.

It is felt that coupled with a base plate, this device will ensure that all the inhaled air is filtered through the HME [5]. In instances of not getting a good seal, a laryngectomy tube that accepts a HME is considered a viable option [5]. The Provox Micron HME is the only device with a virus filter that is available commercially [9].

Along with the HME device, wearing a surgical mask or scarf is always recommended [14].

Therefore, the ideal recommendation is that persons with laryngectomy use facial masks along with filtering devices coupled with a surgical mask for their stomas [11–13, 15]. Those unable to wear an HME due to cost or incompatibility are advised to ensure covering of their stoma with either surgical mask, laryngeal bib, or clothing.

In addition, HMEs need to be disposed appropriately after community exposure [5]. Regular maintenance and cleaning of physical barrier items such as masks, scarfs, and laryngectomy bibs are crucial to ensure protection. Of great importance, laryngectomees need to follow stringent hand washing regimes prior to and immediately following any contact with their stoma [10]. Hand washing with soap or alcohol-based sanitizers needs to be done for a minimum of 20 s [11].

#### Self-protection of home caregiver

Stringent hand hygiene measures also apply to home caregivers of persons with laryngectomy. Direct contact with the tracheostoma (stoma care, voice prosthesis change, inspection of leakage etc.) is regarded as a high-risk procedure [16] because of the increased chances of instigating cough and the aerosolization. Meticulous hand washing prior to and after direct contact is a way to protect themselves and the person with laryngectomy. In addition, it is advisable that the caregiver wears a mask, disposable gloves, and googles while in contact with the stoma [6].

#### Care of devices

It is of utmost importance to follow strict hygiene practices with voice rehabilitation devices considering their close contact with the stoma, mouth, and nose.

- Voice prosthesis

In tracheoesophageal speakers, stomal occlusion by finger for phonation increases the likelihood of virus transmission directly to the trachea in case of contaminated hands [6].

Hand hygiene should be emphasized before and after speaking. Hands-free HME prevents the need for hand occlusion and is therefore beneficial, especially during these pandemic situations [5, 11]. Valves with silver oxide coating and Teflon are recommended to limit biofilm formation [6]. An indwelling device should be cleaned by a dedicated brush. Furthermore, the cleaning brush should be immersed in 70% ethanol or 70% isopropyl alcohol for 10 min or 3% hydrogen peroxide for 60 min as a sanitary measure. Non-indwelling devices should be cleaned by immersing in disinfectants [6].

- Electro larynx
  The electrolarynx should be thoroughly cleaned using an alcohol swab prior to using it for speaking [6].
- Pneumatic device

The pneumatic artificial larynx is more common in Asian countries. This device comprises of a reed which is driven by respiration. One end of the device is cupped over the stoma, whereas the other end comprises of a tube that is placed in the mouth. The vibrations are transferred to the mouth and voicing is produced by movement of the articulators.

The article by Yeung and colleagues recommends cleaned of the tubing by inserting tissue strips as the top and bottom end of the device can be soiled by saliva and sputum respectively. In addition, the device must be immersed in 3% hydrogen peroxide solution for 5–10 min and rinsed with saline or boiled drinking water before use [6].

### Guidelines for clinicians

#### Precautionary measures

Judicious use and knowledge of appropriate personal protection equipment are mandatory to prevent the transmission of viruses and protection of health care workers.

Tracheostoma care, voice prosthesis changing, endoscopy of the upper airway, etc. are regarded as high-risk procedures, with greater chances of spreading aerosols [5, 16].

Enhanced PPE is recommended while caring for laryngectomized patients who are COVID-positive [5, 10, 17] while carrying out procedures with aerosol-generating procedures [5, 10]. Enhanced PPE kit comprising of N95/FFP3/FFP2 mask with a face shield or a powered air-purifying respirator (PAPR), along with gown, gloves [5, 12, 13, 16] disposable caps, and shoe covers, is advisable [5]. Use of a PAPR is strongly recommended, especially while carrying out procedures that require airway manipulation [5, 17].

Minimum of a mask with face shield, gown, and gloves must be worn while in contact with persons with laryngectomy and COVID negative status. However, full PPE is more advisable [13]. In addition, any procedure that has a probability of instigating cough such as stoma care, voice prosthesis changing, and leakage inspection must be done with a COVID-positive assumption and donned in full PPE [5, 13].

Due care has to be taken to ensure that the N95 mask is worn properly without any leakage [10]. Along with the use of appropriate PPEs, it is of utmost importance that clinicians are trained in donning, doffing, and discarding the PPE in a safe manner [10, 16].

### Trouble shooting of device issues during COVID-19 pandemic

#### Management at home

Persons with laryngectomy are advised to remain at home as much as possible [18]. Hospitals have high risks for transmitting the virus, and hence, visits to the hospital should be reserved only for urgent and complicated issues.

Users of voice prosthesis are more likely to require assistance for the management of leakage-related issues. Initial trouble shooting at home is advisable; cleaning the prosthesis thoroughly to ensure full valve closure can be done as a primary step [5]. Temporary use of a TE plug is suggested in cases of leakage through the prosthesis [5]; additionally, dietary modifications such as thickening liquids can also be effective in combating leaks [5, 10, 16].

Other strategies such as using the voice prothesis insertor as a temporary plug and/or holding a cotton tip applicator at the site of leak to absorb drops of liquid are also suggested for use, when necessary [13]. Alternating solid and liquid food items during consumption can also temporarily alleviate leakage. In the case of dislodgment of the voice prosthesis, use of a rubber catheter is advisable before seeking medical help. The rubber catheter can additionally serve the purpose of enteral nutrition if required [5]. Counseling the patient regarding self-management by prosthetic plugs [17] and dietary changes is a viable option when immediate changing of voice prosthesis is not possible. Use of appropriate and accessible augmentative and alternative communication (AAC) or artificial larynx can be recommended temporarily, in case the voice prosthesis fails [19].

#### Telerehabilitation and telemanagement

The use of remote triaging and telesupport for persons with laryngectomy has gained popularity as ways to reduce the exposure of patients to the hospital setting. Simultaneously, these alternatives also ensure that patients do not suffer complications such as leakage-related issues and or cancer recurrence due to deferring of periodic visits. An initial screening through telephone call has been reported in hospitals dealing with this patient population [13].

A prospective cohort study [11] used a remote triaging approach to manage tracheoesophageal speakers using emails, phone calls, and video calls via skype or WhatsApp. These modalities were used to evaluate the patient’s overall condition and resolve issues. Video calls were carried out to counsel patients regarding precautionary measures for protection of the stoma and lower airway. Any problems related to the voice prosthesis were discussed over these video calls. Minor issues that can be managed by the patient were resolved. Only complicated and emergency issues were directed for hospital visit. The results of this study showed that in more than half of the patients, the video call was successful in resolving the problems.

Two cases of tumor recurrence were identified during the video consultations. In both cases, a scheduled hospital visit followed with biopsy and computed tomography scan revealed re-occurrence.

Telerehabilitation has several merits such as limiting hospital visit and thereby limiting physical exposure of an individual, reducing crowding in hospitals, and preservation of PPE. Moreover, this enables clinicians in quarantine to work distantly [20].

However, telerehabilitation can present additional challenges for persons with laryngectomy.

Besides the need for owning a gadget that supports video calls, adequate patient proficiency in using the gadget is also required for successful telerehabilitation. The basic eligibility criteria for telerehabilitation are adequate hearing, vision, and stability to remain in front of the camera [16]. All patients may not be suitable candidates for this approach. Moreover, the alaryngeal speech modes by virtue of their characteristics may impose difficulty of comprehension by the listeners and could be challenging during telecalls.

Involving the patient’s caregiver in teleconsultations could alleviate the communication barrier [19]. Providing the patient with recorded educational video material such as on stoma care and management along with home delivery of the necessary accessories have been suggested as potential ways to enhance telerehabilitation practice [21].

It needs to be emphasized that physical examination offered via telemedicine may not always be ideal, especially for oncology patients who are more susceptible to reoccurrences [19]. It could, however, help in reducing the number of direct hospital visits in the current pandemic situation. A structured hybrid approach that combines teleconsultations and direct consultations could be a viable option and worth exploration in the future. If found suitable, it would be cost-effective for patients, especially those who need periodic hospital visits.

#### Hospital visit

In the current situation, the risk of exposure to covid virus outweighs the benefit of hospital visit. Therefore, the clinical jurisdiction of the clinician would play a pivotal role in making case-by-case judgment. Issues that need immediate attention need to be prioritized over those that can wait or postponed.

A person using tracheoesophageal speech may come across several TEP management issues that require recurrent hospital visits. Though initial home management may temporarily solve the issue, it may not always be wise to hold on for a long time. In such instances, hospital visits are unavoidable and should be done with due caution and adherence to safety measures.

The protocols for hospital visits in the identified articles are variable, attributed to the differences in the density of the pandemic in the respective countries and the individual hospital policies.

Different strategies for patient triaging have been mentioned in the articles. The article by Hennessey presents a useful decision algorithm in handling voice prosthesis-related complications during the pandemic [5]. It is recommended to address instances such as the dislodgement of the voice prosthesis with urgency and use of relevant PPE gear, with the assumption of the patient being positive for COVID-19. Endoscopy procedures could be substituted by imaging techniques such as X rays of chest, abdomen, and CT scan. Patients who are functionally stable and manifest minor issues such as minimal leakage or a dislodged but non-aspirated voice prosthesis can be advised home management strategies until hospital visit is feasible following a negative COVID test result.

Lambertoni described a triaging model to identify Head and neck cancer patients who are in need of direct examination rather than a virtual consultation. They proposed three clinical parameters—"surgical outcome, psychological attitude, oncological risk, and technical skills—based on which a multidisciplinary team identified patient’s candidacy for telemedicine on a weekly basis [19]. Patients who are at a higher risk for recurrence, poorer medical outcomes, psychological issues, or those not well versed with digital device use are directed for face-to-face consultation. Individuals who received the virtual consultation were often accompanied by a caregiver. Guidelines regarding stoma care, managing dysphagia were given along with a physical assessment. The patient feedbacks were also taken using dedicated questionnaires to understand their satisfaction with the telemodality and enhancing it.

The inter-professional care pathway for the management of individuals with laryngectomy at the Princess Margaret cancer center during the pandemic has presented a useful algorithm [13].The model comprised of a collaborative management by speech language pathologist and Head and neck surgeon. All patients were screened primarily by the SLP via a phone call and necessary assistance given for home management of issue, say guidance in ordering supplies, management of leakage issues by cyclic ingestion of solid and liquid foods, use of plugs etc. A video call was arranged for those whose issues could not be solved over the phone. Patients whose issues could not be resolved with a virtual consultation were deferred for a direct consultation. The patients in the deferral list continued to receive guidance via phone calls once in 2 weeks. Those who were called for direct consultation mandatorily underwent covid screening swab (swabs taken from the trachea, oropharynx, and nasopharynx) 24 to 48 h prior. Individuals with covid negative reports were scheduled for a direct consultation wherein the SLP changed the voice prosthesis donned in full PPE. Individuals who tested positive for COVID-19 were required to self-isolate and deferred for later assistance. Those that required urgent attention underwent direct trouble shooting by a head and neck surgeon in full PPE with the addition of PAPR and negative pressure room.

The article by Kligerman and colleagues outlined the guidelines for outpatient and inpatient management of persons with laryngectomy. Individuals who were SARS-CoV2 positive were deferred outpatient visits and those for who deferring was not possible were admitted and attended to by clinicians in aerosolized PPE kits [10]. SARS-COV2 negative patients were advised to wear an HME over the stoma followed by covering it with a surgical mask. Procedures such as stoma care and voice prosthesis change were done with the clinician dressed in aerosolization PPE and procedures that did not involve direct contact with the stoma were done while the health provider donned on droplet PPE.

Overall, the management of voice prosthesis-related issues and stoma care was regarded as high-risk procedures owing to the high possibility of viral transmission through oral and respiratory secretions. It is hence important that prior to out-patient consultations, persons with laryngectomy were administered COVID test. Furthermore, it is advised to carry out this testing 24 to 48 h prior to the voice prosthesis changing procedure [13].

Both esophageal and tracheoesophageal speakers are capable of aerosolization of tracheal secretions and stand to a high risk of transmission of the SARCOV-2 virus. Moreover, instrumental procedures such as nasoendoscopy and tracheoscopy are potential aerosol-generating procedures. In these cases, proper testing of the patient prior to consultation could enable the providers to adequately equip themselves with the appropriate personal protective equipment as a safety measure. Use of enhanced PPEs is required while changing the voice prosthesis [11]. During outpatient consultation, nasopharyngoscopy and tracheoscopy were advised only if deferring was not possible. When performing tracheoscopy, additional care is needed to minimize coughing. Use of lidocaine and or oxymetazoline pledges is advisable if anesthesia is needed. The scope should be immediately cleaned to prevent contamination of counter tops. In the case of dislodged TEP, radiographic imaging tools could be used instead of tracheoscopy or bronchoscopy. After consultation, it is important to sanitize the room and provide adequate time interval for the aerosols to settle before the next use [5].

### Laryngectomees with SARS-COV2 virus

#### COVID testing and manifestations in persons with laryngectomy

In persons with laryngectomy, the upper airway is isolated from the lower respiratory tract. The nasal airflow is minimal when compared to the airflow through the tracheostoma. However, the nasal cavity and sinuses are not free from developing infections.

Of great importance is a case report of a person with a total laryngectomy who had a positive COVID-19 diagnostic test result from a nasopharyngeal swab and a contrary negative result from a tracheal swab [22]. This report warrants mandatory swabbing and analysis from both nose and stoma when carrying out COVID-19 testing in persons with total laryngectomy. The presence of the virus in the upper airway can still transmit the disease considering the potential aerosolization of esophageal and tracheoesophageal speech [22].

Coleman reported four post-laryngectomy patients who tested positive and were managed without intensive care admissions [23]. All four patients presented with COVID infection during the initial peak between March to May 2020. All patients recovered following conservative ward-based care of 2 weeks comprising of oxygen therapy and nebulization along with stoma care.

The case report by Paderno and colleagues presented two patients with laryngectomy who manifested COVID-19 infection in March 2020. Both patients presented with oxygen desaturation thereby requiring bubble-humidified oxygen administration via a mask over the stoma. HME device compatible to the high oxygen flow rate was not available for use at that juncture. Furthermore, both presented with tracheal crusting, for which laryngeal toileting was required.

One patient’s pulmonary health progressively deteriorated leading to demise following 8 days of hospitalization. On the contrary, the other patient remained clinically stable and was discharged following 24 days of hospitalization [24].

Gallo and colleagues described the case of a person with laryngectomy, whose nasal and oropharyngeal swabs were suggestive of SARS-cov2 virus and later confirmed by the endotracheal swab during hospitalization. He received intensive care and ventilatory support for 5 days and improved following 22 day hospital day and moved to a long-term care center [25]. Findings from the case reports are summarized in Table 3.

Hence, COVID testing of persons with laryngectomy must mandatorily include swabs from the nasopharynx and the trachea. Due to the anatomical alterations, reduced immunity due to cancer treatment, and possible likelihood of smoking history, individuals with laryngectomy are at high risk for developing pulmonary complications due to the virus, However, the reports, which are majorly of successful recoveries, are promising.

## Critical appraisal of the studies included

The studies included in the review were compiled in order to present a comprehensive view of the global scenario in managing persons with laryngectomy during the COVID-19 pandemic. Majority of the articles included in the review were commentaries [5, 6, 10, 13, 21], editorials [9,12,,14,18], and letters to the editor [19, 26]. Others were case reports [22–25] or review articles [15–17, 27]. One of the articles was a prospective cohort study [11]. All articles give directions towards the various aspects of immediate management of persons with laryngectomy during this pandemic.

The patient triaging pathways for the management of laryngectomy patients requiring hospital visit or consultations [5, 10, 13, 19] is comprehensive and useful. It offers lucid insights for practicing clinicians. Two of the included articles offered an international and multidisciplinary compendium of tracheostomy care guidance [15] and dysphagia care guidelines [16] to be followed during this COVID crisis. The prospective cohort study on telemedicine for voice prosthesis users offered a look into the scope of telerehabilitation in the future [11].

Two of the case reports were from Italy [24, 25] and one each from Scotland [23] and the USA [22].

In this review, a total of 8 cases of persons with laryngectomy who contracted the SARs-cov-2 virus are reported, out of which 7 recovered. While these reports exude optimism, there is a need to tread with caution with a global perspective of the endemic. Considering the medical vulnerability of this patient group and the likelihood of respiratory complications owing to virus, there is a possibility that many would have succumbed. Across the globe, the front-line medical fraternity is overworked with the successive COVID-19 waves and their manifestation in the respective countries. In the future, when the pandemic diminishes, we may come across more case reports and clinician’s recounts of how they managed persons with laryngectomy during this crisis.

The commentary by Yeung and team [6] is the only one from Asia and the article from Brazil [14] is the only insight from a developing country. The articles give directions on the immediate plan of action for managing laryngectomees in the hospital or provide insights from the management of those who tested positive for COVID-19. There is a glaring lacuna of information from economically backward countries; insights about the clinical and care strategies from these countries would expand our understanding about how individuals with laryngectomy are managed globally during this pandemic and would serve as a ready reckoner in management.

## Discussion

The presence of the stoma with no filtering ability of its own potentially places persons with laryngectomy more vulnerable to receive the SARS COV-2; the structurally intact nasopharynx has the possibility of housing the virus; moreover, esophageal or tracheoesophageal speech can generate aerosolized droplets containing the virus, thereby increasing the risk to the care provider. Therefore, persons with laryngectomy can be considered of high risk or vulnerable to the COVID-19 virus and being more susceptible to transmit the virus to others.

Hand hygiene practices, social distancing, and wearing the mask are the three golden rules to be followed during this pandemic. In persons with laryngectomy, wearing the HME device, preferably with a virus filter and covering the stoma with a surgical mask could be considered an additional self-protection strategy.

Regular maintenance and cleaning of the devices—voice prosthesis, artificial larynx, and pneumatic devices as well as the barrier devices—masks, laryngectomy bib, scarfs, etc. need to be adhered to regularly.

Most of the articles suggested the use of food thickeners to manage minor leakage-related issues and the use of plugs in their voice prosthesis or catheters to temporarily address the leakage. However, placement of catheter may interfere with the stoma care and use of HME and thereby increasing the mucous and affecting pulmonary health, requiring further management. Therefore, the long-term use of a catheter is not advisable, and replacement of voice prosthesis is considered a better option [13]. In addition, removal of a voice prosthesis and insertion of a catheter in essence leaves the patient without a voice, even though temporarily. This is bound to have a psychological effect on the patient. Hence, clinicians should guide the patients and caregivers in using alternative augmentative communication and use of artificial larynx as a standby [27].

Telerehabilitation has gained momentum globally during this pandemic. Though it was seldom used for laryngectomy patients before, the acceptability of this practice has depicted the possible use of it even in the post-pandemic clinical practices. Some of the barriers to telerehabilitation in persons with laryngectomy are communication breakdown due to the poor quality of the alaryngeal speech via phone and incompetency in using the gadgets. However, this issue can be resolved by including a caregiver in the telerehabilitation process. The identification of a recurrence of the tumor in 2 patients in the study has demonstrated the advantages of the telerehabilitation. Longobardi and team recommended the use of a phone camera rather than a computer camera for reasons that close-up images of the oral cavity and tracheostoma can be obtained by using a mobile as opposed to a computer [11]. In addition, they also recommended the involvement of a caregiver/family member during the telematic interaction. The use of telerehabilitation in alaryngeal speakers needs to be further explored even post-pandemic with its probable benefits.

Clinicians must use appropriate PPEs when managing persons with laryngectomy. Changing voice prosthesis, examination of stoma, suctioning, etc. are regarded as high-risk procedures that could generate aerosol droplets. Therefore, these procedures must be done in isolated rooms with strict adherence to the hospital protocol for disinfecting the room before seeing the next patient. Use of innovative strategies such as glass or acrylic barriers is cost-effective option that can help preserve PPEs [26]. However, the efficacy of such modifications needs exploration.

COVID diagnostic testing of individuals with laryngectomy should be done in both the nasopharynx and the trachea. Clinicians should use standard PPEs while handling laryngectomy patients who test negative for COVID-19. Patients with positive or unknown status of COVID-19 should always be examined using enhanced PPE. Regardless of the test status, all aerosolization procedures should be done with enhanced PPE gearing. The summary of the abovementioned guidelines are summarized and presented in Table 2.

The case reports of individuals with laryngectomy have reported issues such as tracheal crusting and lack of availability of accessories compatible with the HME for oxygen delivery at.

Due to the structure altered airway with limited humidification function, persons with laryngectomy more likely to develop tracheal crusting especially when oxygen therapy is required [24].

Periodic tracheal toileting on a regular basis is suggested as an effective way to counter this, prior to the development of severe crusting; in addition, the clinician must be wearing appropriate PPE considering the risk involved with the close contact with the stoma.

The recovery of majority of the patients from the case reports and the fact that most of them did not require intensive care are encouraging.

Majority of the articles included in this review are from developed countries where the scope of telerehabilitation is superior to developing countries. The patients in the less-income countries may not own electronic gadgets that permit video calls, and moreover, even those who have such devices may face additional barriers such as poor mobile network, especially in rural areas.

Majority of the articles have recommended using the HME device over the stoma and if possible, to discard it scientifically after use in crowded places. However, these accessories are often considered a luxury in less developed countries [14] and are seldom used by patients. The cost of HME and incompatibility with humid weather may be two probable reasons for its reduced usage. The lack of knowledge of laryngectomy as a disability in the health policies may also be a factor for the reduced use of HME devices.

Hence, there is a need for reports from third world countries for garnering more information on the management of laryngectomy patients during this global crisis. Furthermore, in the near future, there is a need for more longitudinal studies providing details of the long-term implications of COVID-19 in persons with laryngectomy.

At the time of writing this article, the trajectory of this pandemic is still uncertain with the second wave unleashing its wrath. Deferring hospital visits by the persons with laryngectomy fearing the spread may not hold good for a long time. At this instance, the future is bleak and whether or not all domains of our lives would return to pre-COVID, functioning unknown. It is hereby suggested that the insights received from this pandemic should be carried forward and incorporated into routine clinical management of laryngectomy patients. Telerehabilitation of laryngectomy patients needs to be explored to its fullest potential as it may offer viable options for the management of this medically fragile population.
